# A comparative study of five telerehabilitation therapies for improving core symptoms in stroke patients: A network meta-analysis (2,833 patients)

**DOI:** 10.1016/j.isci.2026.115774

**Published:** 2026-04-17

**Authors:** Yi Xia, Sihan Jin, Weijie Zhou, Rui Huang, Shangjun Huang

**Affiliations:** 1 School of Athletes Performance, Shanghai University of Sport, Shanghai, China; 2 School of Exercise and Health, Shanghai University of Sport, Shanghai, China; 3 Department of Orthopedics and Traumatology, Seventh People’s Hospital of Shanghai University of Traditional Chinese Medicine, Shanghai, China; 4 Department of Rehabilitation, Seventh People’s Hospital of Shanghai University of Traditional Chinese Medicine, Shanghai, China

**Keywords:** Health sciences

## Abstract

This network meta-analysis demonstrates that virtual reality therapy exhibits significant advantages in specific functional domains of remote rehabilitation: Remote virtual reality technology demonstrated the most pronounced effects in improving gait (SUCRA = 92.4%, standardized mean difference [SMD] = −1.27) and upper limb functional recovery (SUCRA = 71.3%, SMD = −0.64), while remote brain-computer interfaces showed the most significant effects in fine motor control. SMD = −1.27) and upper limb functional recovery (SUCRA = 71.3%, SMD = −0.64), while remote brain-computer interfaces showed the greatest effect in fine motor control (SUCRA = 87.6%, SMD = −1.20). Regarding quality of life improvement, exoskeleton training yielded the best results (SUCRA = 62.4%, SMD = 0.05). The findings of this study provide evidence-based support for developing personalized telerehabilitation protocols tailored to specific rehabilitation goals in clinical practice. This approach facilitates a shift in the telerehabilitation field from empirical selection to precision-targeted intervention strategies.

## Graphical abstract

**Figure:**
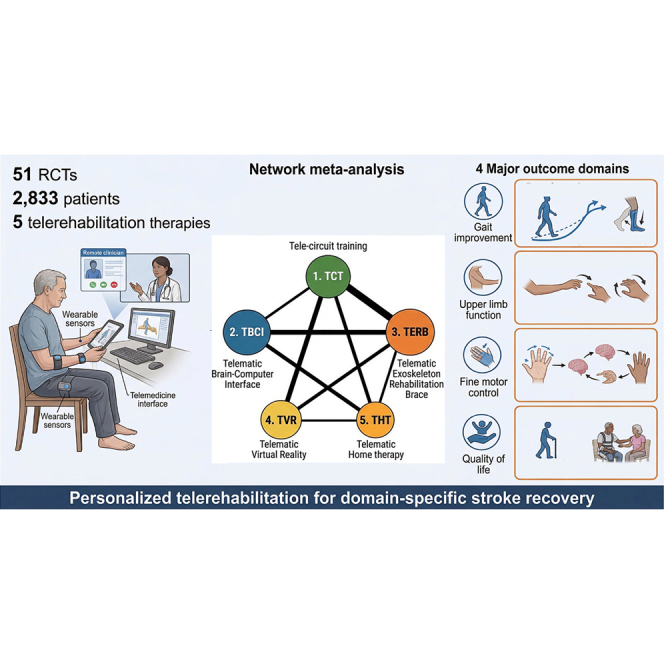

## Introduction

Stroke ranks among the leading causes of disability and death worldwide.^1^ Its high incidence, mortality, and disability rates pose a persistent and severe challenge to public health systems.^2^ With improvements in acute care, patient survival rates have increased. However, approximately two-thirds of survivors often experience varying degrees of physiological dysfunction, such as impairments in motor, sensory, speech, and swallowing functions, severely limiting their ability to perform daily activities and live independently.^3^ These functional impairments not only directly reduce an individual’s physical health status but also trigger psychological issues like anxiety and depression, further diminishing their overall quality of life (QOL).^4^ Moreover, post-stroke functional recovery is a protracted and complex process involving neuroplastic remodeling and multisystem compensation, placing high demands on rehabilitation resource allocation and long-term care.^5^

Without timely rehabilitation intervention, stroke patients may enter a pathophysiological chain leading to poor outcomes.^6^ First, secondary functional impairments worsen.^7^ Poor lower limb motor control leads to loss of walking ability or fixation of abnormal gait patterns, thereby increasing fall risk.^8^^,^^9^ Without intervention, upper limb functional limitations may progress to secondary complications such as muscle atrophy, joint contractures, and intractable pain, significantly complicating subsequent rehabilitation.^10^^,^^11^ Second, the “golden window” for neuroplasticity may be missed.^12^ The brain exhibits its strongest potential for functional reorganization and compensation in the early post-injury period. Without sufficient external stimulation and task-oriented training, the brain’s cortical functional representation areas undergo detrimental remodeling, leading to chronic and permanent functional impairment.^13^^,^^14^ Patients become heavily dependent on others for daily living activities while facing heightened recurrence risks, resulting in diminished QOL and substantially increased familial and socioeconomic burdens.^15^^,^^16^

Stroke is an acute cerebrovascular disorder caused by sudden blockage or rupture of cerebral blood vessels, leading to brain tissue damage.^17^ Primary contributing factors include hypertension, atherosclerosis, heart disease, and unhealthy lifestyles.^18^ These factors can disrupt cerebral blood flow or cause hemorrhage, resulting in localized neurological deficits.^19^ Stroke is characterized by high incidence, high disability rates, and high mortality, often leaving sequelae such as motor impairments, language disorders, and cognitive dysfunction, severely diminishing patients’ QOL.^20^

The primary rehabilitation methods for stroke patients include traditional rehabilitation therapy and physical therapy, which play a crucial role in enhancing activities of daily living.^21^ However, traditional rehabilitation models also present limitations: ensuring sustained treatment adherence after discharge remains challenging, and timely rehabilitation guidance with personalized adjustments is often inadequate. This is particularly true for patients with mobility issues or residing in remote areas, who face practical difficulties in returning to the hospital for regular follow-up appointments. These factors may hinder the continuity and optimization of rehabilitation outcomes. Therefore, developing more practical and convenient intervention models for stroke patient rehabilitation is essential.

Telemedicine, as a product of modern medicine and information technology integration, demonstrates significant advantages in stroke rehabilitation management. It effectively overcomes geographical and transportation barriers, ensuring the continuity and accessibility of rehabilitation training.^22^ Previous stroke rehabilitation research has primarily focused on in-facility interventions, which often suffer from poor service accessibility, low patient compliance, and difficulties in sustaining long-term outcomes. Leveraging information and communication technology, telemedicine overcomes temporal and spatial constraints, enabling patients to receive professional, continuous rehabilitation guidance from home. It not only enhances the coverage and efficiency of rehabilitation services but also facilitates the development of systematic, individualized long-term management plans, offering a viable pathway to improve functional outcomes for stroke patients.^23^

This study employed a network meta-analysis approach to synthesize evidence from five distinct telerehabilitation modalities, evaluating their relative efficacy in improving core stroke symptoms. These symptoms were assessed across four key functional domains: gait, upper limb function (ULF), fine motor control (FMC), and QOL. Notably, the study encompassed specific emerging technologies such as telemedicine brain-computer interface (TBCI) and telemedicine exoskeleton rehabilitation brace (TERB), which had not been comprehensively compared in previous syntheses. By synthesizing direct and indirect evidence across multiple interventions, this analysis aims to address critical gaps in the literature—earlier reviews often exhibited scope limitations, focusing on narrower technology categories, fewer functional outcomes, or smaller datasets.^24^ In contrast, this study incorporates a broader range of technology types and more comprehensive outcome measures to establish a reliable efficacy ranking for remote rehabilitation approaches. This network meta-analysis addresses specific clinical and methodological gaps by synthesizing direct and indirect evidence across multiple interventions. Clinically, it enables cross-comparisons between interventions not directly tested in head-to-head trials, revealing the relative efficacy of emerging telerehabilitation strategies versus established approaches. Methodologically, the study simultaneously evaluates broader technology types and more comprehensive patient-centered outcome measures within a unified analytical framework. The findings aim to establish a reliable efficacy ranking system for telerehabilitation models, providing evidence-based guidance for clinical practice, rehabilitation resource allocation, and the development of personalized telerehabilitation services for stroke survivors.

## Results

### Study selection

**Figure 1:**
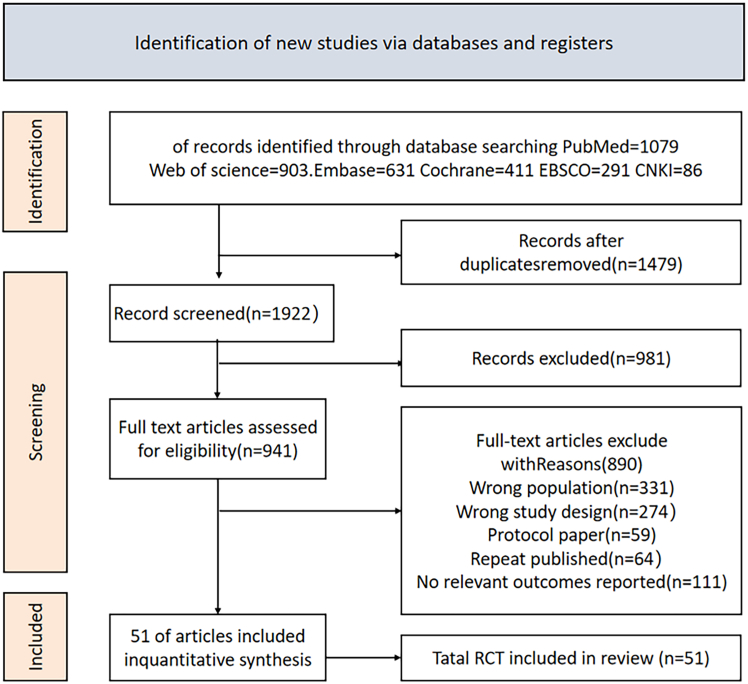
Literature search flowchart

A total of 3,401 potentially relevant studies were identified through literature screening. After removing 1,479 duplicates, 1,922 studies remained for further screening. Title and abstract screening eliminated 981 studies. Based on the inclusion criteria, a comprehensive review and full-text reading of the remaining 941 studies resulted in the exclusion of 890 studies, ultimately including 51 studies. The literature screening flowchart is shown in Figure 1.

### Literature characterization

Literature feature analysis ultimately included 51 studies, with a comprehensive list of their basic characteristics detailed in Table S1. This systematic review and network meta-analysis encompassed studies published between 2000 and 2024 in the United States, the Netherlands, Mexico, China, South Korea, Japan, Spain, Italy, Turkey, the United Kingdom, and Pakistan. The experimental groups comprised 244 stroke patients receiving TCT intervention, 293 receiving TBCI intervention, 324 receiving TERB intervention, 231 receiving TVR intervention, and 294 receiving THT intervention (Table 1). The control group included 1,447 participants, with ages ranging from 40 to 80 years, totaling 2,833 subjects. Table S1 details the characteristics of the included studies. Across 51 randomized controlled trials, we compared five telemedicine therapies (TCT, TBCI, TERB, TVR, and THT) against control groups. TVR was the most frequently used intervention (25.39%), followed by TERB (23.51%) and TBCI (21.56%). Each intervention session lasted 20–120 min. The average duration of exercise intervention was 5.7 weeks (range: 2–24 weeks), with an average of 5.1 exercise sessions per week.

**Table 1:** Operational definitions of five telemedicine interventions

Intervention methods	Abbreviation	Definition
Tele-circuit training	TCT	this is a structured program for circuit training conducted in a home environment with remote guidance^25^
Telematic brain-computer interface	TBCI	by integrating brain-computer interface and electronic information technology, patients can now receive brain-computer interface therapy at home^26^
Telematic exoskeleton rehabilitation brace	TERB	using IoT-assisted exoskeleton robots with remote guidance and monitoring as a home-based program for stroke patients^27^
Telematic virtual reality	TVR	a movement intervention method integrating virtual reality technology with remote monitoring, enabling at-home training^28^
Telematic home therapy	THT	refers to a rehabilitation training model primarily conducted at the patient’s residence under remote guidance^29^

### Risk of bias assessment

**Figure 2:**
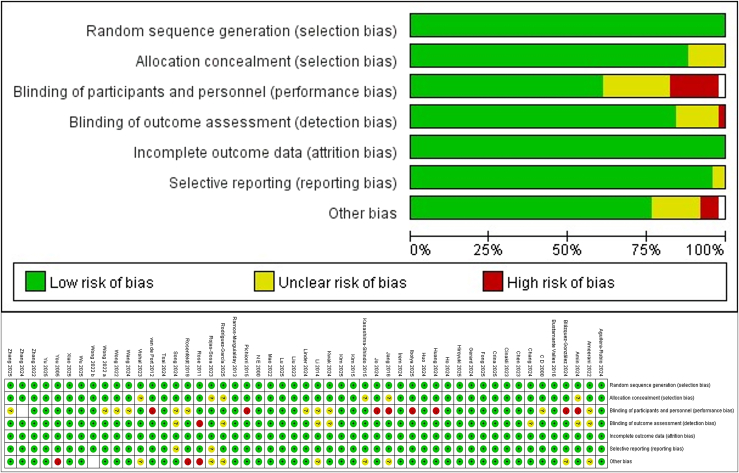
Risk assessment results

Table S2 and Figure 2 detail the risk of bias assessments for each study. Among the 51 studies, all 51 involved a random allocation scheme: 32 described the random allocation method in detail, and 4 discussed allocation concealment strategies; 19 reported blinding implementation, and 44 reported outcome assessment blinding; 49 studies showed a low risk of selective reporting; No other biases were identified across all studies. Overall, the risk of bias across all 51 studies was assessed as low risk of bias (ROB).

### Direct pairwise meta-analyses (primary outcome)

#### Gait improvement

**Figure 3:**
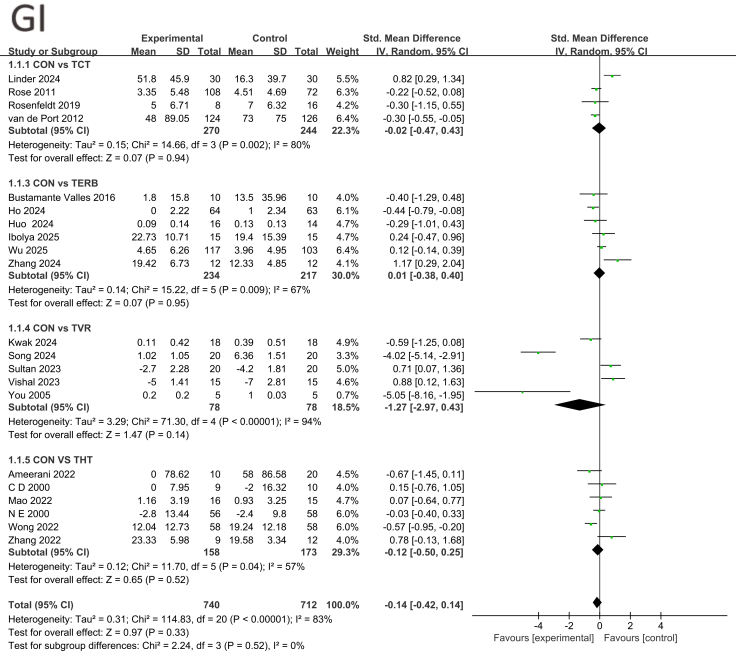
Forest plot for each pairwise comparison of GI; SMDs for each individual study are represented by squares, with size reflecting statistical weight; horizontal lines denote 95% CIs Diamonds represent pooled SMDs for each subgroup and the overall analysis.

This study systematically evaluated the effects of various telemedicine interventions on gait improvement (GI) by incorporating multiple randomized controlled trials involving 1,452 participants. Lower SMD values indicate greater benefit. Figure 3 presents the results of a forest plot analysis comparing each intervention with the control group. In subgroup analyses, the pooled effect size comparing CON with TCT was SMD = −0.02 (95% confidence interval [CI]: −0.47, 0.43; *p* = 0.94), showing no significant difference but high heterogeneity (I^2^ = 80%), indicating substantial methodological or population variability across studies. The pooled effect size for CON versus TBCI was SMD = 0.01 (95% CI: −0.38, 0.40, *p* = 0.95). Although not statistically significant, some studies like Zhang (2024) showed substantial positive effects (SMD = 1.17), suggesting TBCI may hold potential in certain contexts. The effect size comparing CON with TVR was SMD = −1.27 (95% CI: −2.97, 0.43, *p* = 0.14). Although not significant, studies by Song (2024) and You (2005) demonstrated strong negative effects, indicating substantial variability in TVR intervention outcomes across studies with high heterogeneity (*I*^2^ = 94%). In summary, current evidence indicates that various GI interventions in telemedicine do not demonstrate overall significant superiority over conventional training. Although some studies suggest potential for TBCI and TVR, their effects remain unstable due to high inter-study heterogeneity. See Figure 3 for details.

#### FMC

**Figure 4:**
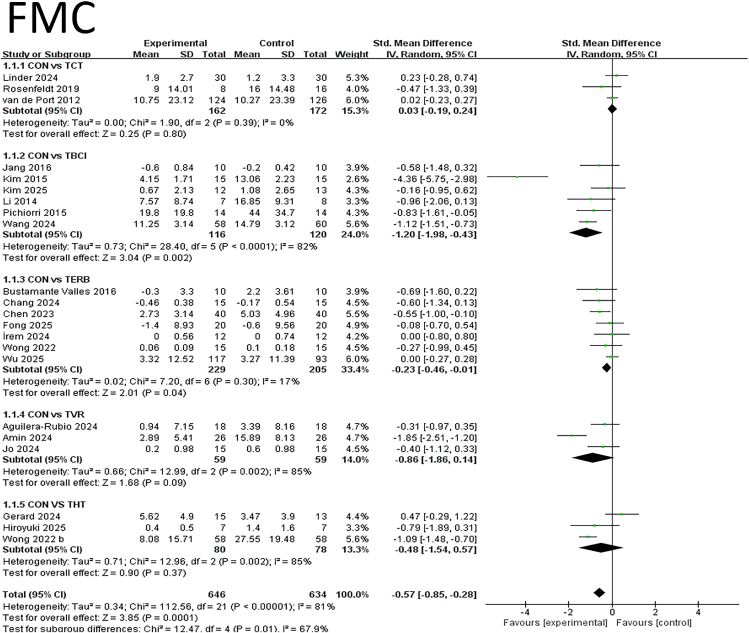
Forest plot for each pairwise comparison of FMC; SMDs for each individual study are represented by squares, with size reflecting statistical weight; horizontal lines denote 95% CIs Diamonds represent pooled SMDs for each subgroup and the overall.

This meta-analysis evaluated the impact of different interventions on fine motor function, incorporating multiple studies. Lower SMD values indicate greater benefit. Figure 4 presents the forest plot analysis results for fine motor function. Analysis revealed that TBCI training demonstrated the most significant improvement among all interventions, with a standardized mean difference (SMD) of −1.20 (95% CI: −1.98, −0.43; *p* = 0.002). This effect size was the largest and highly statistically significant, indicating a marked advantage of TBCI in enhancing fine motor skills. Additionally, TEBR demonstrated a positive effect with an SMD of −0.23 (95% CI: −0.46, 0.01; *p* = 0.04), which, though smaller, remained statistically significant. In contrast, TCT, TVR, and THT showed no significant differences compared to the control group (*p* > 0.05). Notably, TVR training exhibited high heterogeneity (*I*^2^ = 85%), suggesting potential methodological or participant characteristic differences across studies. The overall effect size for THT was −0.48 (95% CI: −1.54, 0.57; *p* = 0.37), failing to reach statistical significance. Subgroup analysis revealed significant between-group heterogeneity (Chi^2^ = 12.47, *p* = 0.01, *I*^2^ = 87.9%), indicating distinct effects of different intervention types on fine motor function. The overall effect test was significant (*Z* = 3.85, *p* = 0.0001), supporting the positive impact of telemedicine interventions on fine motor function. In summary, TBCI training emerged as the most effective telerehabilitation intervention for improving fine motor function, demonstrating significant and superior outcomes compared to other approaches. While TERB showed some effect, its magnitude was limited. Conventional rehabilitation, virtual reality, and human-computer interaction training did not reveal significant advantages in this analysis. See Figure 4 for details.

#### ULF

**Figure 5:**
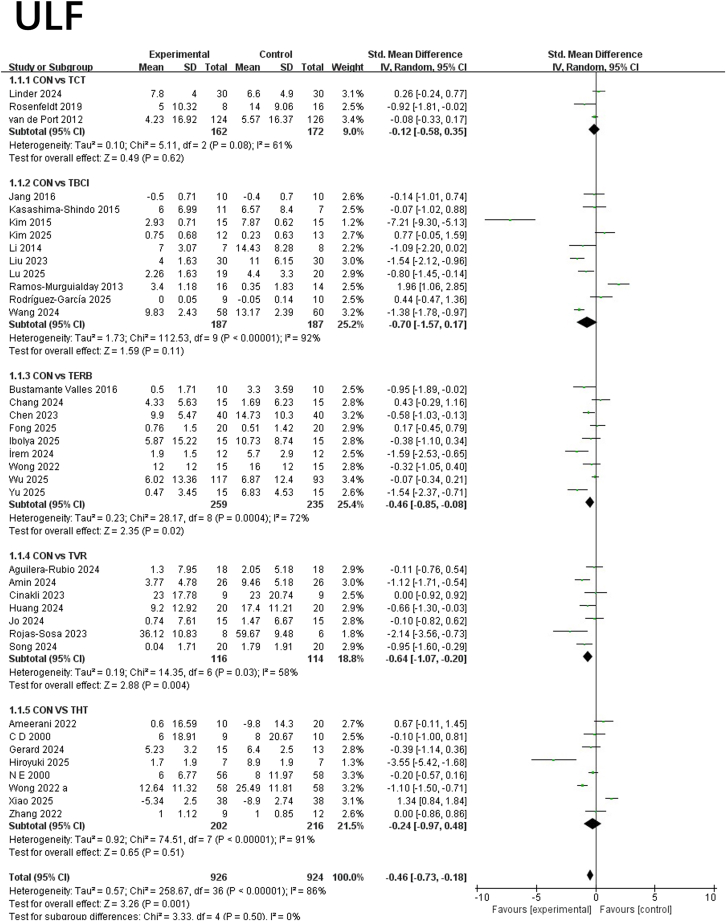
Forest plot for each pairwise comparison of ULF; SMDs for each individual study are represented by squares, with size reflecting statistical weight; horizontal lines denote 95% CIs Diamonds represent pooled SMDs for each subgroup and the overall.

This study conducted a meta-analysis on the effects of different interventions in telemedicine on improving ULF. Lower SMD values indicate greater benefit. Analysis revealed that TERB demonstrated significant advantages in improving ULF. Compared with the control group, TBCI showed a SMD of −0.46 (95% CI: −0.85, −0.08; *p* = 0.02), indicating a moderate effect size with statistical significance and moderate heterogeneity (*I*^2^ = 72%). TVR also demonstrated significant effects, with a SMD of −0.64 (95% CI: −1.07, −0.20; *p* = 0.004). Its effect size was slightly larger than that of TBCI, with moderate heterogeneity (*I*^2^ = 58%). Brain-computer interfaces showed a trend toward improvement (SMD = −0.70, 95% CI: −1.57, 0.17) but failed to reach statistical significance (*p* = 0.11), with high heterogeneity (*I*^2^ = 92%), indicating substantial variation in effect across studies. TCT showed no significant difference compared to the control group (SMD = −0.12, *p* = 0.62), with a small effect size and moderate heterogeneity (*I*^2^ = 61%). Due to incomplete data in some studies, the overall effect of THT remains unclear; however, individual studies demonstrated large effect sizes, suggesting potential efficacy. In summary, both TERB and TVR demonstrated significant effects in improving ULF, with TVR exhibiting the largest effect size and good statistical stability. While brain-computer interfaces show potential, results are unstable with high heterogeneity, requiring cautious interpretation. TCT did not show clear advantages in this analysis. See Figure 5 for details.

#### QOL

**Figure 6:**
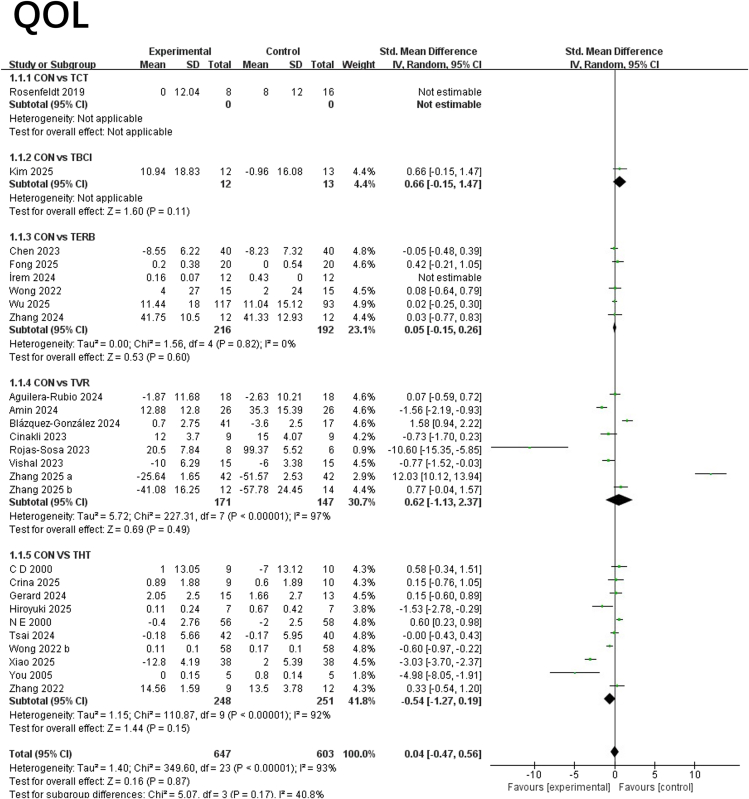
Forest plot for each pairwise comparison of forest of QOL; SMDs for each individual study are represented by squares, with size reflecting statistical weight; horizontal lines denote 95% CIs Diamonds represent pooled SMDs for each subgroup and the overall.

This study systematically compared the effects of five interventions on improving QOL, incorporating 1,250 participants from multiple studies. Lower SMD indicates impairment. As shown in Figure 6, the forest plot meta-analysis revealed an overall effect size of 0.04 (95% CI: −0.47, 0.56; *p* = 0.87), indicating no significant difference in quality-of-life improvement between any intervention and the control group. In subgroup analyses, TBCI showed a modest positive trend (Std. MD = 0.66, 95% CI: −0.15, 1.47; *p* = 0.11), though it did not reach statistical significance. The effect size for TERB intervention was 0.05 (95% CI: −0.15, 0.26; *p* = 0.60), suggesting similar efficacy to the control group. This group exhibited low heterogeneity (*I*^2^ = 0%) and consistent results across studies. The effect size for TVR intervention was 0.62 (95% CI: −1.13, 2.37; *p* = 0.49), but with extremely high heterogeneity (*I*^2^ = 97%), indicating significant variation in outcomes across studies, potentially influenced by intervention content, device type, or population characteristics. The effect size for THT was −0.54 (95% CI: −1.27, 0.19; *p* = 0.15), showing a slight tendency toward the control group but also failing to reach statistical significance. High heterogeneity (*I*^2^ = 92%) was present, suggesting that implementation methods or patient adherence may contribute to inconsistent outcomes. Overall, this meta-analysis of RCTs interventions found no significant superiority of any telemedicine approach over controls in improving QOL. While some interventions like TBCI and TVR showed potential, their effects remain unclear and are subject to substantial heterogeneity. See Figure 6 for details.

### Network meta-analysis

#### Network diagram of included studies

**Figure 7:**
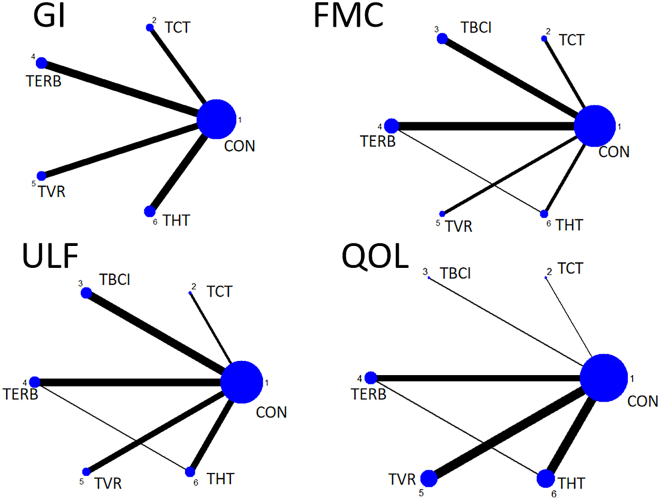
Network plot of outcome indicators

Figure 7 presents the NMA network diagram for four telemedicine therapies, used to evaluate the effectiveness of five interventions. Node size reflects the sample size of each therapy, while line thickness indicates the number of studies comparing these interventions. Among them, TVR is the most commonly used method, while TCT has fewer studies. Figure 7 details the network diagram of outcome measures.

#### Ranking of intervention effectiveness of nine exercise therapy

##### GI indicators

**Figure 8:**
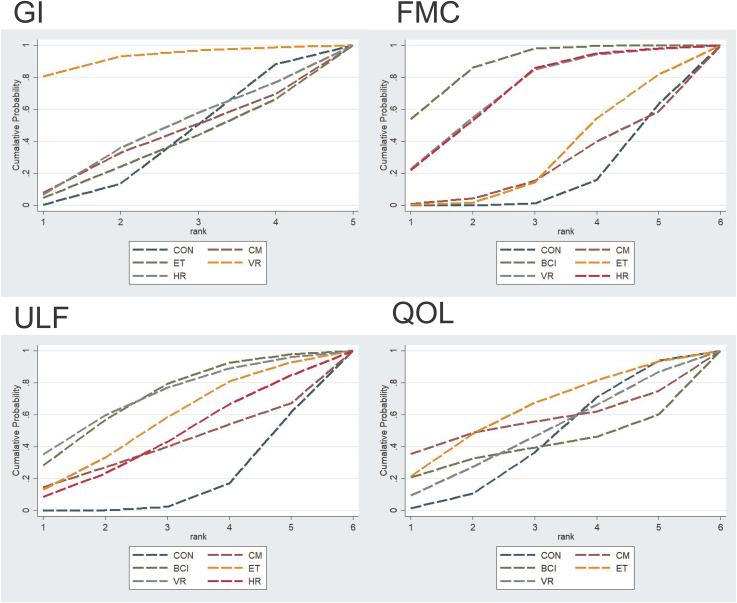
Area under the curve for cumulative ranking probability

Effectiveness ranking of four telemedicine therapies for GI in stroke patients: TVR (SUCRA = 92.4), HR (SUCRA = 44.3), TCT (SUCRA = 40.3), CON (SUCRA = 38.2), and TBCI (SUCRA = 34.8). See Figure 8 and Tables 2, and 3 for details.

**Table 2:** Ranking the probability of nine exercise therapy

Treatment	Gait improvement		Treatment	Fine motor control		Treatment	Upper limb function		Treatment	Quality of life	
	SUCRA (%)	Rank		SUCRA (%)	Rank		SUCRA (%)	Rank		SUCRA (%)	Rank
CON	38.2	4	CON	16.1	6	CON	16.2	6	CON	42.7	5
TCT	40.3	3	TCT	23.9	5	TCT	40.6	5	TCT	55.4	2
TERB	34.8	5	TBCI	87.6	1	TBCI	70.9	2	TBCI	39.9	6
TVR	92.4	1	TERB	30.5	4	TERB	55.7	3	TERB	62.4	1
THT	44.3	2	TVR	71.0	2	TVR	71.3	1	TVR	47.3	4
–	–	–	THT	70.9	3	THT	45.2	4	THT	52.4	3

**Table 3:** Network meta-analysis matrix of outcome

**Gait improvement**					

TVR	−0.92 (−2.29,0.45)	−0.98 (−2.46,0.49)	−0.99 (−2.05,0.07)	−1.04 (−2.41,0.32)	–
0.92 (−0.45,2.29)	THT	−0.06 (−1.41,1.28)	−0.06 (−0.93,0.80)	−0.12 (−1.34,1.10)	–
0.98 (−0.49,2.46)	0.06 (−1.28,1.41)	TCT	−0.00 (−1.03,1.03)	−0.06 (−1.40,1.28)	–
0.99 (−0.07,2.05)	0.06 (−0.80,0.93)	0.00 (−1.03,1.03)	CON	−0.06 (−0.91,0.80)	–
1.04 (−0.32,2.41)	0.12 (−1.10,1.34)	0.06 (−1.28,1.40)	0.06 (−0.80,0.91)	TERB	–

**Fine motor control**					

TBCI	−0.32 (−1.47,0.83)	−0.32 (−1.47,0.82)	−1.00 (−1.91,−0.09)	−1.13 (−2.26,−0.00)	−1.18 (−1.87,−0.48)
0.32 (−0.83,1.47)	TVR	−0.01 (−1.30,1.28)	−0.69 (−1.78,0.40)	−0.81 (−2.09,0.47)	−0.86 (−1.78,0.06)
0.32 (−0.82,1.47)	0.01 (−1.28,1.30)	THT	−0.68 (−1.69,0.33)	−0.80 (−2.08,0.47)	−0.85 (−1.76,0.06)
1.00 (0.09,1.91)	0.69 (−0.40,1.78)	0.68 (−0.33,1.69)	TERB	−0.13 (−1.19,0.94)	−0.17 (−0.76,0.41)
1.13 (0.00,2.26)	0.81 (−0.47,2.09)	0.80 (−0.47,2.08)	0.13 (−0.94,1.19)	TCT	−0.05 (−0.94,0.84)
1.18 (0.48,1.87)	0.86 (−0.06,1.78)	0.85 (−0.06,1.76)	0.17 (−0.41,0.76)	0.05 (−0.84,0.94)	CON

**Upper limb function**					

TVR	−0.02 (−1.18,1.14)	−0.24 (−1.40,0.92)	−0.37 (−1.57,0.83)	−0.47 (−2.05,1.11)	−0.69 (−1.57,0.19)
0.02 (−1.14,1.18)	TBCI	−0.21 (−1.28,0.85)	−0.35 (−1.46,0.76)	−0.44 (−1.96,1.07)	−0.66 (−1.42,0.09)
0.24 (−0.92,1.40)	0.21 (−0.85,1.28)	TERB	−0.13 (−1.20,0.93)	−0.23 (−1.74,1.28)	−0.45 (−1.20,0.30)
0.37 (−0.83,1.57)	0.35 (−0.76,1.46)	0.13 (−0.93,1.20)	THT	−0.10 (−1.64,1.44)	−0.32 (−1.13,0.50)
0.47 (−1.11,2.05)	0.44 (−1.07,1.96)	0.23 (−1.28,1.74)	0.10 (−1.44,1.64)	TCT	−0.22 (−1.53,1.09)
0.69 (−0.19,1.57)	0.66 (−0.09,1.42)	0.45 (−0.30,1.20)	0.32 (−0.50,1.13)	0.22 (−1.09,1.53)	CON

**Quality of life**					

TERB	−0.11 (−7.28,7.06)	−0.43 (−3.72,2.86)	−0.67 (−4.18,2.84)	−0.75 (−3.41,1.90)	−1.41 (−8.57,5.75)
0.11 (−7.06,7.28)	TCT	−0.32 (−7.33,6.68)	−0.56 (−7.60,6.48)	−0.64 (−7.30,6.01)	−1.30 (−10.71,8.11)
0.43 (−2.86,3.72)	0.32 (−6.68,7.33)	THT	−0.24 (−3.40,2.93)	−0.32 (−2.50,1.86)	−0.98 (−7.98,6.02)
0.67 (−2.84,4.18)	0.56 (−6.48,7.60)	0.24 (−2.93,3.40)	TVR	−0.08 (−2.37,2.21)	−0.74 (−7.77,6.29)
0.75 (−1.90,3.41)	0.64 (−6.01,7.30)	0.32 (−1.86,2.50)	0.08 (−2.21,2.37)	CON	−0.66 (−7.31,5.99)
1.41 (−5.75,8.57)	1.30 (−8.11,10.71)	0.98 (−6.02,7.98)	0.74 (−6.29,7.77)	0.66 (−5.99,7.31)	TBCI

##### FMC indicator

Ranking of five telemedicine therapies for FMC: TBCI (SUCRA = 87.6%), TVR (SUCRA = 71.0%), THT (SUCRA = 70.9%), TERB (SUCRA = 30.5%), TCT (SUCRA = 23.9%), and CON (SUCRA = 16.1%). See Figure 8 and Tables 2 and 3 for details.

##### ULF indicator

Ranking of five telemedicine therapies for upper limb functional recovery: TVR (SUCRA = 71.3%), TBCI (SUCRA = 70.9%), TBCI (SUCRA = 55.7%), THT (SUCRA = 45.2%), TCT (SUCRA = 40.6%), and CON (SUCRA = 16.2%). See Figure 8 and Tables 2 and 3 for details.

##### QOL indicators

The ranking of five telemedicine therapies for patient QOL was as follows: TBCI (SUCRA = 62.4%), TCT (SUCRA = 55.4%), THT (SUCRA = 52.4%), TVR (SUCRA = 47.3%), CON (SUCRA = 42.7%), and TBCI (SUCRA = 39.9%). See Figure 8 and Tables 2 and 3 for details.

### Ranking of intervention effects for outcome indicators

In terms of GI, the TVR group (SUCRA = 92.4%, ranked first) and the THT group (SUCRA = 44.3%, ranked second) demonstrated more pronounced improvements than the control group (CON), with the TVR group showing particularly outstanding results. The TCT group (SUCRA = 40.3%, ranked third) was marginally superior to the control group, while the TBCI group (SUCRA = 34.8%, ranked fifth) demonstrated relatively weaker effects. Overall, TVR intervention showed the greatest potential for GI, THT also exhibited some positive effects, whereas TERB had more limited efficacy. See Table 2 for details.

Regarding FMC, SUCRA rankings indicated that TBCI intervention yielded the best outcomes (SUCRA = 87.6%, ranked first), followed by TVR (SUCRA = 71.0%, ranked second) and THT (SUCRA = 70.9%, ranked third), all significantly outperforming the control group (CON). TERB (SUCRA = 30.5%, fourth rank) and TCT (SUCRA = 23.9%, fifth rank) showed relatively weaker effects, while the control group (CON) had the lowest SUCRA value (16.1%, sixth rank), indicating the smallest improvement. In summary, TBCI, TVR, and THT demonstrate superior therapeutic potential for improving FMC. See Table 2 for details.

Regarding ULF, the network meta-analysis revealed that the TVR group (SUCRA = 71.3%, ranked first) and TBCI group (SUCRA = 70.9%, ranked second) significantly outperformed the control group (CON), with TVR showing the most pronounced improvement followed by TBCI. The TBCI group (SUCRA = 55.7%, ranked third) and THT group (SUCRA = 45.2%, ranked fourth) also demonstrated moderate improvement effects. The TCT group (SUCRA = 40.6%, ranked fifth) was marginally superior to the control group, though the improvement was limited. The control group (CON) had the lowest SUCRA value (16.2%, ranked sixth), indicating its relatively weak efficacy in restoring ULF. See Table 2 for details.

Regarding QOL, based on the cumulative ranking probability curve area (SUCRA) results, the effectiveness of each intervention was ranked as follows: TERB group (SUCRA = 62.4%, ranked first), TCT group (SUCRA = 55.4%, ranked second), THT group (SUCRA = 52.4%, ranked third), TVR group (SUCRA = 47.3%, fourth), control group (CON) (SUCRA = 42.7%, fifth), and TBCI group (SUCRA = 39.9%, sixth). Among these, TBCI, TCT, and THT demonstrated superior QOL improvement compared to the control group, while TVR and TBCI showed relatively weaker effects. See Table 2 for details.

### Publication bias and small sample size tests

**Figure 9:**
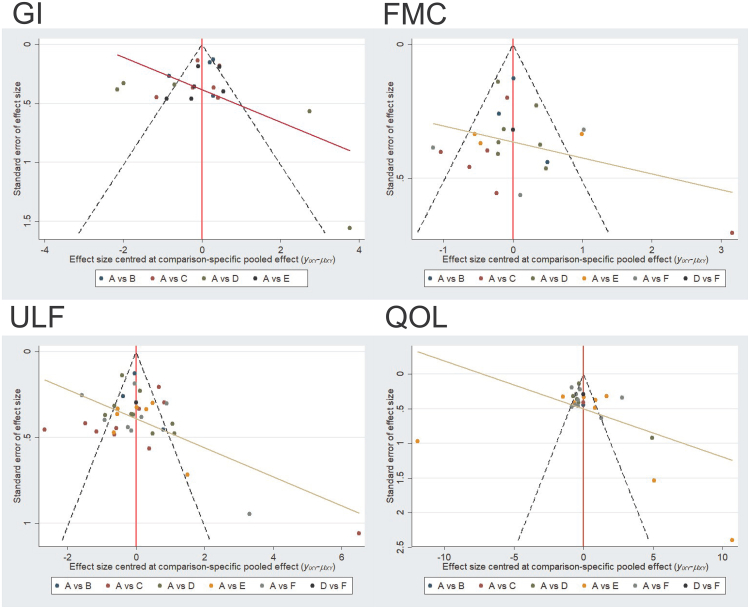
Comparison funnel plot for outcome indicators; A = TCT, B = TBCI, C = TERB, D = TVR, E = THT

In the studies included in the network meta-analysis, adjusted comparison funnel plots were used for small-sample effect size estimation and publication bias testing. The included studies were largely symmetrical; specific details are shown in Figure 9.

## Discussion

### Effects on GI

Regarding GI in stroke patients, TVR showed the highest cumulative ranking probability (SUCRA = 92.4%), suggesting it may be the most promising intervention; however, its pooled effect size was not statistically significant (*p* = 0.14). This indicates that while TVR ranks favorably among the compared interventions, its superiority remains uncertain within the current evidence. Theoretically, TVR immersive and task-oriented design may enhance engagement and promote motor recovery, but high heterogeneity (*I*^2^ = 94%) across studies, likely due to variations in VR protocols and patient characteristics, limits conclusive interpretation. As reflected in conflicting results reported in studies such as Sana (2023) and Laver (2017), this highlights the necessity for enhanced standardization of TVR interventions to improve consistency.^30^^,^^31^ In contrast, TCT did not demonstrate significantly superior outcomes compared to conventional care and exhibited similarly high heterogeneity (*I*^2^ = 80%), potentially attributable to variations in training intensity and individual adherence. TERB similarly failed to demonstrate significant overall efficacy.^32^ Although individual studies like Zhang (2024) suggested potential benefits, existing evidence remains insufficient to support TERB as the preferred option for gait rehabilitation. In summary, while telerehabilitation holds immense clinical potential for gait recovery in stroke patients, its application requires rigorous attention to protocol standardization and individualized adaptive adjustments.^33^

### Effects on motor control

For FMC, the TBCI was identified as the most effective intervention, ranking highest in efficacy. This finding is consistent with prior research suggesting that TBCI facilitates the reorganization of the motor cortex through closed-loop neural feedback, often integrated with functional electrical stimulation or visual cues, thereby specifically enhancing the coordination and accuracy of fine hand movements.^34^ TVR and THT followed in ranking; however, their comparative effects did not reach statistical significance against the control group, and both exhibited considerable heterogeneity across studies. This heterogeneity likely arises from differences in VR task design for TVR and variability in patient adherence, family support, and remote guidance quality for THT. In contrast, the TERB demonstrated only limited efficacy for fine motor recovery. Overall, TBCI shows the greatest promise as a telerehabilitation strategy for improving FMC post-stroke, supported by its superior ranking and mechanistic alignment with neuroplasticity principles.

### Effects on upper limb functional

In terms of upper limb functional recovery, TVR and TBCI have been demonstrated as the most promising telerehabilitation interventions. This aligns with previous research findings indicating that for stroke patients with motor impairments, telerehabilitation yields outcomes comparable to—and in some cases superior to—in-person therapy.^35^ Following TVR intervention, significant improvements were observed in upper limb coordination, motor function perception, and shoulder flexion and abduction range of motion, with statistically significant differences in shoulder abduction range of motion changes.^36^ Although TVR and TBCI show positive trends, their high heterogeneity warrants cautious interpretation. TERB demonstrated moderate rehabilitation efficacy, while TCT and THT yielded only limited benefits—consistent with earlier findings that low-intensity or unstructured home-based rehabilitation programs yield poorer outcomes for upper limb functional recovery.^37^ Overall, television-based rehabilitation and remote training represent cutting-edge technological strategies for enhancing ULF, supporting their integration into personalized rehabilitation programs.

### Effects on QOL

Regarding QOL improvement, TERB and TCT demonstrated the highest potential among the evaluated telemedicine interventions. This observation aligns partially with previous research suggesting structured physical activity can enhance QOL indirectly through functional and psychological gains.^38^ In contrast, interventions such asTVR and TBCI showed more limited efficacy for QOL outcomes. The impact of TVR was characterized by high heterogeneity, which likely reflects substantial variation in intervention protocols, device characteristics, and patient factors, extending earlier findings that its benefits may be domain-specific and moderated by multiple variables.^39^ Similarly, THT presented moderate potential but with considerable heterogeneity, indicating that its effectiveness is highly dependent on factors such as patient adherence and the quality of remote support. Overall, TERB and TCT appear to offer more consistent promise for enhancing QOL, whereas advanced technological interventions like TVR and TBCI may require further personalization and integrative strategies to optimize their impact in this domain.

### A discussion of traditional rehabilitation

Technology-based interventions show potential in improving specific motor functions (such as gait, ULF, and FMC) in stroke patients. Their effects may surpass traditional rehabilitation therapies, particularly for chronic-phase stroke patients requiring high-repetition task training and long-term home-based rehabilitation maintenance. However, this advantage is conditional: technology-based interventions have not demonstrated consistent or clear superiority in improving QOL, acute/subacute phase application, or universal applicability.^40^ Their efficacy is generally moderate and significantly influenced by the standardization of intervention protocols and patient heterogeneity. Most direct comparative studies have yielded effect sizes that fail to reach statistical significance. Specifically, Chen et al. found that remote home-based rehabilitation significantly improved Fugl-Meyer scores for both upper and lower limbs in stroke patients compared to traditional rehabilitation, demonstrating positive effects on motor function.^41^ However, Mulder’s study showed no significant difference in functional activity capacity between the telerehabilitation and traditional rehabilitation groups.^42^ This discrepancy may stem from differences in participant characteristics and intervention duration. Chen’s study primarily recruited subcortical stroke patients for a 12-week intervention, whereas Mulder’s study focused on subacute-phase (≤3 months) patients receiving an 8-week intervention without restricting stroke type or location. Therefore, the advantages of technological interventions should be viewed as complementary or optimizing measures within specific contexts, rather than comprehensive replacements for traditional rehabilitation.^43^ Future research should prioritize achieving precise matching of patient characteristics, standardizing intervention protocols, and conducting high-quality controlled studies to integrate the strengths of both technological and traditional rehabilitation approaches.

### VR technology adoption

Patient acceptance, ease of use, and satisfaction are key factors determining the successful implementation and long-term sustainability of telerehabilitation. Studies indicate that stroke patients demonstrate high acceptance of telerehabilitation exercise systems and express strong willingness to use them.^44^^,^^45^ Although some patients experienced minor adverse reactions during intervention, they generally found the technology enjoyable.^46^ Overall, patient satisfaction with telerehabilitation is comparable to that of traditional in-person rehabilitation.^47^ Furthermore, telerehabilitation systems offer high learnability and masterability due to their gamified exercise experiences.^48^

### Limitations

Despite the comprehensive nature of this network meta-analysis, several important limitations remain. First, substantial heterogeneity existed across studies, particularly regarding GI and quality-of-life outcomes (e.g., *I*^2^ = 94%–97%). This may stem from variations in intervention protocols—such as training duration, frequency, intensity, and applied techniques—as well as patient characteristics including stroke onset time, baseline functional status, and comorbidities. This limits the generalizability of findings and underscores the need for enhanced standardized reporting and subgroup analyses in future studies. Second, although funnel plots did not indicate significant publication bias, selective reporting of results remains possible due to sample size disparities and a preference for positive outcomes; additionally, excluding non-English and non-Chinese literature may introduce language bias. Third, practical and technical limitations were not systematically evaluated. The high cost and limited accessibility of advanced equipment may constrain practical application and healthcare equity. Technical issues and significant learning curves for both clinicians and patients could impact intervention efficacy. Furthermore, the risk of cyber sickness induced by virtual reality may reduce tolerance among certain populations. Fourth, the lack of standardization across platforms and devices complicates comparative efficacy assessments and clinical replication. Finally, analyses have focused on short-to-medium-term outcomes (2–24 weeks), leaving long-term efficacy, sustainability of benefits, and post-intervention adherence unclear. Furthermore, evidence for specific patient subgroups (e.g., those with severe cognitive impairment or very elderly patients) remains insufficient, limiting conclusions about broad applicability. Future research should incorporate longer follow-up periods, standardized intervention protocols, and individualized patient analyses. Conducting practical studies to evaluate cost-effectiveness, accessibility, usability, and safety across diverse clinical populations will provide more robust guidance for clinical practice.

## Acknowledgments

This study was supported by Science and Technology Development Fund of Shanghai Pudong New Area (KJW2024-Y18), Medical Engineering Cross Innovation Special Project of Shanghai Seventh People's Hospital (QYYGZ0202), Traditional Chinese Medicine Peak Discipline Construction Project of Pudong New Area Health Commission (YC-2023-0601), Shiyinyu National Famous Traditional Chinese Medicine Inheritance Studio (YC-2023-0120), Key Department Construction Project of Collaborative Traditional Chinese and Western Medicine, Discipline Construction Project “Rehabilitation Clinical Medicine Center” of Seventh People’s Hospital of 10.13039/501100010876Shanghai University of TCM (25-LCYZX-05), and Discipline Construction Project ‘Intelligent Rehabilitation Engineering’ of Shanghai Pudong New Area Health Commission (PWXx2025-08).

## Author contributions

All authors contributed to the conception and design of the study. Data collection and analysis were performed by Y.X. The first draft of the manuscript was written by Y.X. and S.H., and all authors reviewed and approved the final manuscript.

## Declaration of interests

The authors declare no competing interests.

## Supplemental information

Table S1. Basic features of the included

Table S2. Evaluation results of literature quality risk bias of included articles

## References

[1] Wu S., Wu B., Liu M., Chen Z., Wang W., Anderson C.S., Sandercock P., Wang Y., Huang Y., Cui L. (2019). Stroke in China: advances and challenges in epidemiology, prevention, and management. Lancet Neurol..

[2] Tu W.J., Wang L.D., Special Writing Group of China Stroke Surveillance Report (2023). China stroke surveillance report 2021. Mil. Med. Res..

[3] Nogueira N.G.d.H.M., Parma J.O., Leão S.E.S.d.A., Sales I.d.S., Macedo L.C., Galvão A.C.D.R., de Oliveira D.C., Murça T.M., Fernandes L.A., Junqueira C. (2021). Mirror therapy in upper limb motor recovery and activities of daily living, and its neural correlates in stroke individuals: A systematic review and meta-analysis. Brain Res. Bull..

[4] Guo J., Wang J., Sun W., Liu X. (2022). The advances of post-stroke depression: 2021 update. J. Neurol..

[5] Jones T.A. (2017). Motor compensation and its effects on neural reorganization after stroke. Nat. Rev. Neurosci..

[6] Verma G., Sarmah D., Datta A., Goswami A., Rana N., Kaur H., Borah A., Shah S., Bhattacharya P. (2023). Pharmacological Strategies for Stroke Intervention: Assessment of Pathophysiological Relevance and Clinical Trials. Clin. Neuropharmacol..

[7] Raghavan P. (2015). Upper Limb Motor Impairment After Stroke. Phys. Med. Rehabil. Clin. North Am..

[8] Mehrholz J., Pohl M., Kugler J., Elsner B. (2018). The Improvement of Walking Ability Following Stroke. Dtsch. Arztebl. Int..

[9] Walsh M., Galvin R., Horgan N.F. (2017). Fall-related experiences of stroke survivors: a meta-ethnography. Disabil. Rehabil..

[10] Huang J., Ji J.R., Liang C., Zhang Y.Z., Sun H.C., Yan Y.H., Xing X.B. (2022). Effects of physical therapy-based rehabilitation on recovery of upper limb motor function after stroke in adults: a systematic review and meta-analysis of randomized controlled trials. Ann. Palliat. Med..

[11] Alsubiheen A.M., Choi W., Yu W., Lee H. (2022). The Effect of Task-Oriented Activities Training on Upper-Limb Function, Daily Activities, and Quality of Life in Chronic Stroke Patients: A Randomized Controlled Trial. Int. J. Environ. Res. Public Health.

[12] Dimyan M.A., Cohen L.G. (2011). Neuroplasticity in the context of motor rehabilitation after stroke. Nat. Rev. Neurol..

[13] Dąbrowski J., Czajka A., Zielińska-Turek J., Jaroszyński J., Furtak-Niczyporuk M., Mela A., Poniatowski Ł.A., Drop B., Dorobek M., Barcikowska-Kotowicz M., Ziemba A. (2019). Brain Functional Reserve in the Context of Neuroplasticity after Stroke. Neural Plast..

[14] Crozier J., Roig M., Eng J.J., MacKay-Lyons M., Fung J., Ploughman M., Bailey D.M., Sweet S.N., Giacomantonio N., Thiel A. (2018). High-Intensity Interval Training After Stroke: An Opportunity to Promote Functional Recovery, Cardiovascular Health, and Neuroplasticity. Neurorehabil. Neural Repair.

[15] Gurková E., Štureková L., Mandysová P., Šaňák D. (2023). Factors affecting the quality of life after ischemic stroke in young adults: a scoping review. Health Qual. Life Outcomes.

[16] GBD 2019 Stroke Collaborators (2021). Global, regional, and national burden of stroke and its risk factors, 1990-2019: a systematic analysis for the Global Burden of Disease Study 2019. Lancet Neurol..

[17] Boursin P., Paternotte S., Dercy B., Sabben C., Maïer B. (2018). Sémantique, épidémiologie et sémiologie des accidents vasculaires cérébraux [Semantics, epidemiology and semiology of stroke]. Soins.

[18] Zhao Y., Zhang X., Chen X., Wei Y. (2022). Neuronal injuries in cerebral infarction and ischemic stroke: From mechanisms to treatment (Review). Int. J. Mol. Med..

[19] Hilkens N.A., Casolla B., Leung T.W., de Leeuw F.E. (2024). Stroke. Lancet (London, England).

[20] Bello U.M., Chutiyami M., Salihu D., Abdu S.I., Tafida B.A., Jabbo A.A., Gamawa A., Umar L., Lawan A., Miller T., Winser S.J. (2021). Quality of life of stroke survivors in Africa: a systematic review and meta-analysis. Qual. Life Res..

[21] Minelli C., Luvizutto G.J., Cacho R.d.O., Neves L.d.O., Magalhães S.C.S.A., Pedatella M.T.A., Mendonça L.I.Z.d., Ortiz K.Z., Lange M.C., Ribeiro P.W. (2022). Brazilian practice guidelines for stroke rehabilitation: Part II. Diretrizes brasileiras para reabilitação no acidente vascular cerebral: parte II. Arq. Neuropsiquiatr..

[22] Demaerschalk B.M., Miley M.L., Kiernan T.E.J., Bobrow B.J., Corday D.A., Wellik K.E., Aguilar M.I., Ingall T.J., Dodick D.W., Brazdys K. (2009). Stroke telemedicine. Mayo Clin. Proc..

[23] Hacke W. (2021). Telemedizinische Versorgung beim Schlaganfall [Telemedicine in Stroke Care]. Nervenarzt.

[24] Cumpston M., Li T., Page M.J., Chandler J., Welch V.A., Higgins J.P., Thomas J. (2019). Updated guidance for trusted systematic reviews: a new edition of the Cochrane Handbook for Systematic Reviews of Interventions. Cochrane Database Syst. Rev..

[25] Turkmen C., Karakus A., Yilmaz S., Sengül F., Sigirtmac I.C. (2025). Preliminary Efficacy of Face-to-Face, Telerehabilitation, and Home Program-Based Task-Oriented Circuit Training on the Cognitive and Motor Functions of Older Adults: A Single-Blind Randomized Feasibility Study. Am. J. Occup. Ther..

[26] Mansour S., Giles J., Nair K.P.S., Marshall R., Ali A., Arvaneh M. (2025). A clinical trial evaluating feasibility and acceptability of a brain-computer interface for telerehabilitation in patients with stroke. J. NeuroEng. Rehabil..

[27] Kuo L.C., Yang K.C., Lin Y.C., Lin Y.C., Yeh C.H., Su F.C., Hsu H.Y. (2023). Internet of Things (IoT) Enables Robot-Assisted Therapy as a Home Program for Training Upper Limb Functions in Chronic Stroke: A Randomized Control Crossover Study. Arch. Phys. Med. Rehabil..

[28] Piron L., Turolla A., Tonin P., Piccione F., Lain L., Dam M. (2008). Satisfaction with care in post-stroke patients undergoing a telerehabilitation programme at home. J. Telemed. Telecare.

[29] Chen J., Sun D., Zhang S., Shi Y., Qiao F., Zhou Y., Liu J., Ren C. (2020). Effects of home-based telerehabilitation in patients with stroke: A randomized controlled trial. Neurology.

[30] Sana V., Ghous M., Kashif M., Albalwi A., Muneer R., Zia M. (2023). Effects of vestibular rehabilitation therapy versus virtual reality on balance, dizziness, and gait in patients with subacute stroke: A randomized controlled trial. Medicine.

[31] Laver K.E., Lange B., George S., Deutsch J.E., Saposnik G., Crotty M. (2017). Virtual reality for stroke rehabilitation. Cochrane Database Syst. Rev..

[32] Hacke W. (2021). Telemedizinische Versorgung beim Schlaganfall [Telemedicine in Stroke Care]. Nervenarzt.

[33] Mansour S., Giles J., Nair K.P.S., Marshall R., Ali A., Arvaneh M. (2025). A clinical trial evaluating feasibility and acceptability of a brain-computer interface for telerehabilitation in patients with stroke. J. NeuroEng. Rehabil..

[34] Endzelytė E., Petruševičienė D., Kubilius R., Mingaila S., Rapolienė J., Rimdeikienė I. (2025). Integrating Brain-Computer Interface Systems into Occupational Therapy for Enhanced Independence of Stroke Patients: An Observational Study. Medicina (Kaunas, Lithuania).

[35] Sarfo F.S., Ulasavets U., Opare-Sem O.K., Ovbiagele B. (2018). Tele-Rehabilitation after Stroke: An Updated Systematic Review of the Literature. J. Stroke Cerebrovasc. Dis..

[36] Chen J., Or C.K., Li Z., Yeung E.H.K., Zhou Y., Hao T. (2023). Effectiveness, safety and patients' perceptions of an immersive virtual reality-based exercise system for poststroke upper limb motor rehabilitation: A proof-of-concept and feasibility randomized controlled trial. Digit. Health.

[37] Louie D.R., Mortenson W.B., Durocher M., Schneeberg A., Teasell R., Yao J., Eng J.J. (2021). Efficacy of an exoskeleton-based physical therapy program for non-ambulatory patients during subacute stroke rehabilitation: a randomized controlled trial. J. NeuroEng. Rehabil..

[38] Jiang L., Ding H., Ma Q., Gao S., Zhang X., Chun B. (2025). Comparing the effectiveness of different exercise interventions on quality of life in stroke patients: a randomized controlled network meta-analysis. BMC Neurol..

[39] Dąbrowská M., Pastucha D., Janura M., Tomášková H., Honzíková L., Baníková Š., Filip M., Fiedorová I. (2023). Effect of Virtual Reality Therapy on Quality of Life and Self-Sufficiency in Post-Stroke Patients. Medicina (Kaunas, Lithuania).

[40] Appleby E., Gill S.T., Hayes L.K., Walker T.L., Walsh M., Kumar S. (2019). Effectiveness of telerehabilitation in the management of adults with stroke: A systematic review. PLoS One.

[41] Chen J., Sun D., Zhang S., Shi Y., Qiao F., Zhou Y., Liu J., Ren C. (2020). Effects of home-based telerehabilitation in patients with stroke: A randomized controlled trial. Neurology.

[42] Mulder M., Nikamp C.D., Prinsen E.C., Nijland R.H., van Dorp M., Buurke J., Kwakkel G., van Wegen E.E. (2024). Allied rehabilitation using caregiver-mediated exercises combined with telerehabilitation for stroke (ARMed4Stroke): A randomised controlled trial. Clin. Rehabil..

[43] Appleby E., Gill S.T., Hayes L.K., Walker T.L., Walsh M., Kumar S. (2019). Effectiveness of telerehabilitation in the management of adults with stroke: A systematic review. PLoS One.

[44] Chen J., Or C.K., Li Z., Yeung E.H.K., Chen T. (2025). Perceptions of Patients With Stroke Regarding an Immersive Virtual Reality-Based Exercise System for Upper Limb Rehabilitation: Questionnaire and Interview Study. JMIR Serious Games.

[45] Stephenson A., Howes S., Murphy P.J., Deutsch J.E., Stokes M., Pedlow K., McDonough S.M. (2022). Factors influencing the delivery of telerehabilitation for stroke: A systematic review. PLoS One.

[46] Warland A., Paraskevopoulos I., Tsekleves E., Ryan J., Nowicky A., Griscti J., Levings H., Kilbride C. (2019). The feasibility, acceptability and preliminary efficacy of a low-cost, virtual-reality based, upper-limb stroke rehabilitation device: a mixed methods study. Disabil. Rehabil..

[47] Muñoz-Tomás M.T., Burillo-Lafuente M., Vicente-Parra A., Sanz-Rubio M.C., Suarez-Serrano C., Marcén-Román Y., Franco-Sierra M.Á. (2023). Telerehabilitation as a Therapeutic Exercise Tool versus Face-to-Face Physiotherapy: A Systematic Review. Int. J. Environ. Res. Public Health.

[48] Chen Y., Chen Y., Zheng K., Dodakian L., See J., Zhou R., Chiu N., Augsburger R., McKenzie A., Cramer S.C. (2020). A qualitative study on user acceptance of a home-based stroke telerehabilitation system. Top. Stroke Rehabil..

