# Supplementary material for: A comparative study of five telerehabilitation therapies for improving core symptoms in stroke patients: A network meta-analysis (2,833 patients)
Source: iScience. 2026 Apr 17;29(6):115774. doi: 10.1016/j.isci.2026.115774 (PMC13196438; doi:10.1016/j.isci.2026.115774)
Supplement: Table S1. Basic features of the included [file mmc1.pdf]

iScience, Volume *29*

Supplemental information

A comparative study of five telerehabilitation therapies for improving core symptoms in stroke patients: A network meta-analysis (2,833 patients)

Yi Xia, Sihan Jin, Weijie Zhou, Rui Huang, and Shangjun Huang

Appendix 1. Basic features of the included

Study	Country	Acute/Chronic	group	Sample(M/F)	Age (mean ± SD)	Duration	frequency and duration	Ischemic or infraction/Hemorrhag
Linder 2024[30]	USA	Chronic	CON	30（18/12）	60.8 ± 11.5	8w	45+45min,3days/week	24/6
			TCT	30（17/13）	60.2 ± 9.8		45+45min,3days/week	23/7
Rosenfeldt 2019[31]	USA	Chronic	TCT	16（12/4）	51 ± 12	8w	45+45min,3days/week	
			CON	8（7/1）	58 ± 12		45+45min,3days/week	
van de Port 2012[32]	Netherlands	Acute	TCT	126(82/44)	56±10	12w	90min,2days/week	103/23
			CON	124(80/44)	58±10		90min,2days/week	100/24
Rose 2011[33]	USA	Acute	CON	108（45/63）	68.0 ± 13.1	3w	1.5h,5days/week	
			TCT	72（31/41）	67.6 ± 15.3		1.5h,5days/week	
Rodríguez-García 2025[34]	Mexico	Chronic	TBCI	10（9/1）	50.92±16.82	6w	1h,5days/week	6/4
			CON	9（7/2）	54.56±15.64		1h,5days/week	6/3
Lu 2025[35]	China	Chronic	TBCI	20(17/3)	52.60±14.48	4w	30min,5days/week	
			CON	19(16/3)	50.42±13.61		30min,5days/week	
Kim 2025[36]	Korea	Chronic	CON	12（10/2）	49.0 ± 16.9	4w	1h,5days/week	5/7
			TBCI	13（9/4）	46.0 ± 12.8		1h,5days/week	4/9
Wang 2024[37]	China	Acute	TBCI	150(110/40)	60±11.11	1mo	30min,5days/week	
			CON	146(115/31)	58±10.37		30min,5days/week	
Liu 2023[38]	China	Acute	TBCI	30(22/8)	52.5±10.59	3w	20min,5days/week	22/8
			CON	30(19/11)	53.0±15.56		20min,5days/week	17/13
Kim 2015[39]	USA	Chronic	TBCI	15(6/9)	59.07±8.07	4w	30min,5days/week	
			CON	15(6/9)	59.93±9.79		30min,5days/week	
Jang 2016[40]	Korea	Acute	TBCI	10（6/4）	61.10±13.77	6w	20min,5days/week	6/4
			CON	10（4/6）	61.70±12.09		30min,5days/week	6/4
Kasashima-Shindo 2015[41]	Japan	Chronic	CON	11(9/2)	53.5±12.4	2w	10min,5days/week	6/5
			TBCI	7(4/3)	48.0±9.7		45min,5days/week	3/4
Ramos-Murguialday 2013[42]	Spain	Chronic	TBCI	16（9/7）	49.3 ± 12.5	4w	30min+30min/5days/week	
			CON	14（9/5）	50.3 ± 12.2		1h,5days/week	
Li 2014[43]	China	Acute	TBCI	8(5/4)	67±4.96	8w	1.5h,3days/week	6/2
			CON	7(6/1)	67.14±5.96		5days/week	5/2

Pichiorri 2015[44]	Rome	Acute	CON	14	64.1 ± 8.4	4w	3h，3days/week	12/2
			TBCI	14	59.6 ± 12.7		30min,3days/week	12/2
Yu 2025[45]	China	Acute	CON	15(10/5)	52.18 ±14.41	2w	20min ,5day/week	15(8/7)
			TBCI	15(12/3)	59 ±9.21		30min,5 day/week	15(9/5/1)
Wu 2025[46]	China	Acute	CON	117（80/37）	60.2 ± 13.4	4w	2time×20min ，5day/week	
			TBCI	93(67/26)	59.7 ± 13.4		2time×20min ，5day/week	71/46
Fong 2025[47]	China	Chronic	TBCI	20(12/8)	55.45 ± 9.98	4w	60 min ，5 day/week	u
			CON	20(13/7)	57.80 ± 9.22		60 min ，5 day/week	
Ibolya 2025[48]	Budapest	Acute	CON	15(11/4)	55±11.98	4w	30min ,15 times within 4 weeks	15(14/1)
			TBCI	15(10/5)	57±9.85		30min ,15 times within 4 weeks	15(11/4)
Bustamante Valles 2016[49]	Mexico	Chronic	CON	10(4/6)	44.1 ± 12.55	8W	120 min, 3.4-4.6 days/week	
			TBCI	10(3/7)	64.1 ± 8.38		120 min, 3.4-4.6 days/week	
Chang 2024[50]	Korea.	Chronic	TBCI	15（7/8）	65.2 ± 5.4	4w	30min 4week/10time	15(8/7)
			CON	15(7/8)	67.2 ± 5.4		30min 4week/10time	15(8/7)
İrem 2024[51]		Chronic	TBCI	12(9/3)	57.3 ± 12.1	6w	40-60min，2day/week	12(11/1)
			CON	12(5/7)	66.7 ± 10.5		40-60min，2day/week	12(10/2)
Chen 2023[52]	China	Acute	TBCI	40(34/6)	50.1±10	4w	45min ,5 day/week	40（21/19）
			CON	40(33/7)	53.7±9.4		45min ,5 day/week	40(20/20)
Wong 2022[53]	Norway	Acute	TBCI	15(10/15)	63±9	4w	60min,5 day/week	15（9/6）
			CON	15(13/2)	56±12		60min,5 day/week	15(10/5)
Zhang 2024[54]	China	Acute	TBCI	12（10/2）	63.50±8.97	4w	40min 5day/week	12(5/7)
			CON	12(8/4)	63.83±8.28		60min 5day/week	12(7/5)
Huo 2024[55]	China	Acute	TBCI	14(10/4)	57.93± 11.47	4w	30min，2time/day，5 day/week	14(8/6)
			CON	16(11/5)	55.25± 11.16		30min，2time/day，5 day/week	16(13/3)
Ho 2024[56]	China	Acute	TBCI	63(36/27)	58.73 ± 8.91	12w	60min 3 day/week	63（48/14）
			CON	64(40/24)	56.19 ± 9.55		60min 3 day/week	64(46/18)
Song 2024[57]	China	Acute	TVR	20（12/8）	54.65±7.92	2w	20min,5days/week	
			CON	20（14/6）	55.80±7.55		no report	
Jo 2024[58]	south Korea	Acute	TVR	15（7/8）	51.73±13.63	4w	30min,3days/week	
			CON	15（8/7）	47.13±13.91		60min,5days/week	
Huang 2024[59]	China	Acute	TVR	20(13/7)	63.3±14.3	3w	30min+30min,5days/week	19/1
			CON	20(11/9)	65.1±6.1		60min,5days/week	18/2
Cinakli 2023[60]	Turkey	Chronic	TVR	9（4/5）	61.00±11.48	4w	30min,5days/week	8/1

			CON	9（4/5）	56.00±8.89		40min,5days/week	8/1
Amin 2024[61]	PK	Acute	TVR	26（16/10）	51.8±12.9	6w	24min+24min,4days/week; after four week40min+40min,4days/week	
			CON	26（18/8）	49.8±9.9		24min+24min,4days/week; after four week40min+40min,4days/week	
Aguilera-Rubio 2024[62]	Spain	Chronic	TVR	18（8/10）	60.33 ± 12.44	8w	30min+30min,2days/week	15/3
			CON	18（12/6）	69.56 ± 10.63		60min,2days/week	17/1
Rojas Sosa 2023[63]	Mexico	Acute	TVR	6(2/4)	68.3±2.1	6w	1h,2days/week	
			CON	7(1/6)	66.7±3.6		1h,2days/week	
Zhang 2025[64]	China	Acute		42（24/18）	64.98±9.66	4w	30min,5days/week	35/7
				42（29/13）	66.43±13.12		30min,5days/week	34/8
Blázquez-González 2024[65]	ES	Acute	TVR	17（15/2）	54.23±6.53	6w	20min,1day/week	6/11
			CON	41（22/19）	54.29±7.19		400-600min/week	21/20
Vishal 2023[66]	Pakistan	Acute	CON	15（5/10）	54.87 ± 9.47	8w	30min,3days/week	
			TVR	15（8/7）	49.73 ± 5.81		30min,3days/week	
You 2005[67]	USA	Chronic	TVR	5(4/1)	54.60±3.01	4w	60min,5days/week	2/3
			CON	5(3/2)	54.60±3.44		60min,5days/week	3/2
Kwak 2024[68]	Korea	Chronic	TVR	18(10/8)	54.28±17.74	5w	30min，3days/week	2/16
			CON	18(11/7)	59.17±13.86		30min，5days/week	4/14
Sultan 2023[69]	Pakistan	Chronic	TVR	20(15/5)	58.6±3.3	8w	30min,3days/week	18/2
			CON	20(16/4)	58.1±4.3		30min,3days/week	18/2
Xiao 2025[70]	China	Acute	CON	38（16/22）	56.7 ± 5.3	24w	20min 5time	38/0
			THT	38(21/17)	55.9 ± 6.2		20min 5time	38/0
Hiroyuki 2025[71]	Japan	Chronic	CON	7（6/1）	58.0±6.4	4w	30 min ，5 days/week	7(4/3)
			THT	7(6/1)	51.4±6.7		30 min ，5 days/week	7(1/6)
Gerard 2024[72]	USA	Chronic	CON	15(13/2)	58.00 ±11.1	12w	20 min, every day	U
			THT	13（9/4）	55.87 ±14.5		20 min, every day	
Zhang 2022[73]	China	Chronic	CON	13（12/1）	51.31 ± 8.56	12w	40-60min,1 day/week	13（7/6）
			THT	12(10/2)	53.25 ± 5.75		40-60min,2 day/week	12(8/4)
Wong 2022[74]	China	Acute	THT	58（42/16）	66.21±10.07	12w	90min,5 days/week	58/0
			CON	58(39/19)	67.00±8.61		90min,5 days/week	58/0
Ameerani 2022[75]	Suriname	Chronic	THT	20(9/11)	61.6 ± 9.1	8w	70 min,3 days/week	20(18/2)
			CON	10(4/6)	62.2 ± 9.1		70 min,3 days/week	10(9/1)

C D 2000[76]	UK	Acute	THT	23（10/13	*72±12*	12w	180 min, every day	U
			CON	20(8/12)	*76±7.4*		20min, every day	
N E 2000[77]	Canada	Acute	THT	58（37/11）	70.36 12.7	4w	4day,4week	U
			CON	56(40/16)	69.66 12.7		4day,4week	
Crina 2025[78]	UK	Chronic	THT	10（7/3）	56.5 ±15.76	2w	30min 1day/week	10（7/3）
			CON	8(4/4)	57 ±18.87		30min 1day/week	8(5/3)
Tsai 2024[79]	China	Acute	THT	40（20/20）	59.35±14.08	4w	30min	40(23/17)
			CON	42(29/13)	61.79±14.52		30min	42(12/30/)
Mao 2022[80]	China	Chronic	CON	16（13/3）	52.25 ±9.21	3w	120 min, every day	16/0
			THT	15（12/3）	54.80 ±10.64		120 min, every day	15/0

[1] Linder, S. M., Bischof-Bockbrader, A., Davidson, S., Li, Y., Lapin, B., Singh, T., Lee, J., Bethoux, F., & Alberts, J. L. (2024). The Utilization of Forced-Rate Cycling to Facilitate Motor Recovery Following Stroke: A Randomized Clinical Trial. Neurorehabilitation and neural repair, 38(4), 291–302. https://doi.org/10.1177/15459683241233577

[2] Rosenfeldt, A. B., Linder, S. M., Davidson, S., Clark, C., Zimmerman, N. M., Lee, J. J., & Alberts, J. L. (2019). Combined Aerobic Exercise and Task Practice Improve Health-Related Quality of Life Poststroke: A Preliminary Analysis. Archives of physical medicine and rehabilitation, 100(5), 923–930. https://doi.org/10.1016/j.apmr.2018.11.011

[3] van de Port, I. G., Wevers, L. E., Lindeman, E., & Kwakkel, G. (2012). Effects of circuit training as alternative to usual physiotherapy after stroke: randomised controlled trial. BMJ (Clinical research ed.), 344, e2672. https://doi.org/10.1136/bmj.e2672

[4] Rose, D., Paris, T., Crews, E., Wu, S. S., Sun, A., Behrman, A. L., & Duncan, P. (2011). Feasibility and effectiveness of circuit training in acute stroke rehabilitation. Neurorehabilitation and neural repair, 25(2), 140–148. https://doi.org/10.1177/1545968310384270

[5] Rodríguez-García, M. E., Carino-Escobar, R. I., Carrillo-Mora, P., Hernandez-Arenas, C., Ramirez-Nava, A. G., Pacheco-Gallegos, M. D. R., Valdés-Cristerna, R., & Cantillo-Negrete, J. (2025). Neuroplasticity changes in cortical activity, grey matter, and white matter of stroke patients after upper extremity motor rehabilitation via a brain-computer interface therapy program. Journal of neural engineering, 22(2), 10.1088/1741-2552/adbebf. https://doi.org/10.1088/1741-2552/adbebf

[6] Lu, R., Pang, Z., Gao, T., He, Z., Hu, Y., Zhuang, J., Zhang, Q., & Gao, Z. (2025). Multisensory BCI promotes motor recovery via high-order network-mediated interhemispheric integration in chronic stroke. BMC medicine, 23(1), 380. https://doi.org/10.1186/s12916-025-04214-8

[7] Kim, M. S., Park, H., Kwon, I., An, K. O., Kim, H., Park, G., Hyung, W., Im, C. H., & Shin, J. H. (2025). Efficacy of brain-computer interface training with motor imagery-contingent feedback in improving upper limb function and neuroplasticity among persons with chronic stroke: a double-blinded, parallel-group, randomized controlled trial. Journal of neuroengineering and rehabilitation, 22(1), 1. https://doi.org/10.1186/s12984-024-01535-2

[8] Wang, A., Tian, X., Jiang, D., Yang, C., Xu, Q., Zhang, Y., Zhao, S., Zhang, X., Jing, J., Wei, N., Wu, Y., Lv, W., Yang, B., Zang, D., Wang, Y., Zhang, Y., Wang, Y., & Meng, X. (2024). Rehabilitation with brain-computer interface and upper limb motor function in ischemic stroke: A randomized controlled trial. Med (New York, N.Y.), 5(6), 559–569.e4. https://doi.org/10.1016/j.medj.2024.02.014

[9] Liu, X., Zhang, W., Li, W., Zhang, S., Lv, P., & Yin, Y. (2023). Effects of motor imagery based brain-computer interface on upper limb function and attention in stroke patients with hemiplegia: a randomized controlled trial. BMC neurology, 23(1), 136. https://doi.org/10.1186/s12883-023-03150-5

[10] Kim, T., Kim, S., & Lee, B. (2016). Effects of Action Observational Training Plus Brain-Computer Interface-Based Functional Electrical Stimulation on Paretic Arm Motor Recovery in Patient with Stroke: A Randomized Controlled Trial. Occupational therapy international, 23(1), 39–47. https://doi.org/10.1002/oti.1403

[11] Jang, Y. Y., Kim, T. H., & Lee, B. H. (2016). Effects of Brain-Computer Interface-controlled Functional Electrical Stimulation Training on Shoulder Subluxation for Patients with Stroke: A Randomized Controlled Trial. Occupational therapy international, 23(2), 175–185. https://doi.org/10.1002/oti.1422

[12] Kasashima-Shindo, Y., Fujiwara, T., Ushiba, J., Matsushika, Y., Kamatani, D., Oto, M., Ono, T., Nishimoto, A., Shindo, K., Kawakami, M., Tsuji, T., & Liu, M. (2015). Brain-computer interface training combined with transcranial direct current stimulation in patients with chronic severe hemiparesis: Proof of concept study. Journal of rehabilitation medicine, 47(4), 318–324. https://doi.org/10.2340/16501977-1925

[13] Ramos-Murguialday, A., Broetz, D., Rea, M., Läer, L., Yilmaz, O., Brasil, F. L., Liberati, G., Curado, M. R., Garcia-Cossio, E., Vyziotis, A., Cho, W., Agostini, M., Soares, E., Soekadar, S., Caria, A., Cohen, L. G., & Birbaumer, N. (2013). Brain-machine interface in chronic stroke rehabilitation: a controlled study. Annals of neurology, 74(1), 100–108. https://doi.org/10.1002/ana.23879

[14] Li, M., Liu, Y., Wu, Y., Liu, S., Jia, J., & Zhang, L. (2014). Neurophysiological substrates of stroke patients with motor imagery-based Brain-Computer Interface training. The International journal of neuroscience, 124(6), 403–415. https://doi.org/10.3109/00207454.2013.850082

[15] Pichiorri, F., Morone, G., Petti, M., Toppi, J., Pisotta, I., Molinari, M., Paolucci, S., Inghilleri, M., Astolfi, L., Cincotti, F., & Mattia, D. (2015). Brain-computer interface boosts motor imagery practice during stroke recovery. Annals of neurology, 77(5), 851–865. https://doi.org/10.1002/ana.24390

[16] Yu, Y., Huang, W., Tuerxun, H., Zheng, Y., Su, L., Li, X., & Dou, Z. (2025). Enhanced neuroplasticity and gait recovery in stroke patients: a comparative analysis of active and passive robotic training modes. BMC neurology, 25(1), 239. https://doi.org/10.1186/s12883-025-04226-0

[17] Wu, X., Qiao, X., Xie, Y., Yang, Q., An, W., Xia, L., Li, J., & Lu, X. (2025). Rehabilitation training robot using mirror therapy for the upper and lower limb after stroke: a prospective cohort study. Journal of neuroengineering and rehabilitation, 22(1), 54. https://doi.org/10.1186/s12984-025-01590-3

[18] Toh, F. M., Lam, W. W. T., Cruz Gonzalez, P., & Fong, K. N. K. (2025). Effects of a Wearable-Based Intervention on the Hemiparetic Upper Limb in Persons With Stroke: A Randomized

Controlled Trial. Neurorehabilitation and neural repair, 39(1), 31–46. https://doi.org/10.1177/15459683241283412

[19] Tavaszi, I., Szabó, G., Erdősi, P., Shenker, B., & Fazekas, G. (2025). Application of computerised interactive devices for stroke patients with hemispatial neglect. Komputerizált interaktív eszközök alkalmazása hemispaciális neglectben stroke-betegeknél. Ideggyogyaszati szemle, 78(3-04), 107–121. https://doi.org/10.18071/isz.77.0107

[20] Bustamante Valles, K., Montes, S., Madrigal, M.deJ., Burciaga, A., Martínez, M. E., & Johnson, M. J. (2016). Technology-assisted stroke rehabilitation in Mexico: a pilot randomized trial comparing traditional therapy to circuit training in a Robot/technology-assisted therapy gym. Journal of neuroengineering and rehabilitation, 13(1), 83. https://doi.org/10.1186/s12984-016-0190-1

[21] Chang, J. Y., Chun, M. H., Lee, A., Lee, A., & Lee, C. M. (2024). Effects of training with a rehabilitation device (Rebless®) on upper limb function in patients with chronic stroke: A randomized controlled trial. Medicine, 103(26), e38753. https://doi.org/10.1097/MD.0000000000038753

[22] Akgün, İ., Demirbüken, İ., Timurtaş, E., Pehlivan, M. K., Pehlivan, A. U., Polat, M. G., Francisco, G. E., & Yozbatiran, N. (2024). Exoskeleton-assisted upper limb rehabilitation after stroke: a randomized controlled trial. Neurological research, 46(11), 1074–1082. https://doi.org/10.1080/01616412.2024.2381385

[23] Chen, Z. J., He, C., Xu, J., Zheng, C. J., Wu, J., Xia, N., Hua, Q., Xia, W. G., Xiong, C. H., & Huang, X. L. (2023). Exoskeleton-Assisted Anthropomorphic Movement Training for the Upper Limb After Stroke: The EAMT Randomized Trial. Stroke, 54(6), 1464–1473. https://doi.org/10.1161/STROKEAHA.122.041480

[24] Wong, Y., Li, C. J., Ada, L., Zhang, T., Månum, G., & Langhammer, B. (2022). Upper Limb Training with a Dynamic Hand Orthosis in Early Subacute Stroke: A Pilot Randomized Trial. Journal of rehabilitation medicine, 54, jrm00279. https://doi.org/10.2340/jrm.v54.2231

[25] Zhang, Y., Zhao, W., Wan, C., Wu, X., Huang, J., Wang, X., Huang, G., Ding, W., Chen, Y., Yang, J., Su, B., Xu, Y., Zhou, Z., Zhang, X., Miao, F., Li, J., & Li, Y. (2024). Exoskeleton rehabilitation robot training for balance and lower limb function in sub-acute stroke patients: a pilot, randomized controlled trial. Journal of neuroengineering and rehabilitation, 21(1), 98. https://doi.org/10.1186/s12984-024-01391-0

[26] Huo, C., Shao, G., Chen, T., Li, W., Wang, J., Xie, H., Wang, Y., Li, Z., Zheng, P., Li, L., & Li, L. (2024). Effectiveness of unilateral lower-limb exoskeleton robot on balance and gait recovery and neuroplasticity in patients with subacute stroke: a randomized controlled trial. Journal of neuroengineering and rehabilitation, 21(1), 213. https://doi.org/10.1186/s12984-024-01493-9

[27] Ho, H. J., Wu, L. C., Wu, E. H., Lee, S. F., Lee, T. H., Chiang, S. H., Chen, C. H., Chen, H. Y., Pan, S. J., Chen, Y. W., & WEAR-Stroke Study Group (2024). Improving patient outcomes in acute and subacute stroke using a wearable device-assisted rehabilitation system: a randomized controlled trial. The Journal of international medical research, 52(10), 3000605241281425. https://doi.org/10.1177/03000605241281425

[28] Song, C., Wang, L., Ding, J., Xu, C., Yang, H., & Mao, Y. (2024). Effect of Upper Limb Repetitive Facilitative Exercise on Gait of Stroke Patients based on Artificial Intelligence and Computer Vision Evaluation. Journal of musculoskeletal & neuronal interactions, 24(3), 301–309.

[29] Jo, S., Jang, H., Kim, H., & Song, C. (2024). 360° immersive virtual reality-based mirror therapy for upper extremity function and satisfaction among stroke patients: a randomized controlled trial. European journal of physical and rehabilitation medicine, 60(2), 207–215. https://doi.org/10.23736/S1973-9087.24.08275-3

[30] Huang, Q., Jiang, X., Jin, Y., Wu, B., Vigotsky, A. D., Fan, L., Gu, P., Tu, W., Huang, L., & Jiang, S. (2024). Immersive virtual reality-based rehabilitation for subacute stroke: a randomized controlled trial. Journal of neurology, 271(3), 1256–1266. https://doi.org/10.1007/s00415-023-12060-y

[31] Cinakli, H., Yetisgin, A., Sen Dokumaci, D., & Boyaci, A. (2024). Effects of adding interactive videogames to conventional rehabilitation program on radiological progression and upper extremity motor function in patients with hemiplegic stroke: a preliminary study. Somatosensory & motor research, 41(4), 213–221. https://doi.org/10.1080/08990220.2023.2194401

[32] Amin, F., Waris, A., Syed, S., Amjad, I., Umar, M., Iqbal, J., & Omer Gilani, S. (2024). Effectiveness of Immersive Virtual Reality-Based Hand Rehabilitation Games for Improving Hand Motor Functions in Subacute Stroke Patients. IEEE transactions on neural systems and rehabilitation engineering : a publication of the IEEE Engineering in Medicine and Biology Society, 32, 2060–2069. https://doi.org/10.1109/TNSRE.2024.3405852

[33] Aguilera-Rubio, Á., Alguacil-Diego, I. M., Mallo-López, A., Jardón Huete, A., Oña, E. D., & Cuesta-Gómez, A. (2024). Use of low-cost virtual reality in the treatment of the upper extremity in chronic stroke: a randomized clinical trial. Journal of neuroengineering and rehabilitation, 21(1), 12. https://doi.org/10.1186/s12984-024-01303-2

[34] Rojas-Sosa, M. D. C., Zárate, J. A., de la Rosa-Peña, N., Olvera-Gómez, J. L., Rojano-Mejía, D., Delgado-García, J., & Garduño-Espinosa, J. (2023). Aphasia improvement without logotherapy during motor neurorehabilitation of post-stroke hemiparesis using virtual reality or modified constraint-induced movement therapy: A retrospective cohort. NeuroRehabilitation, 53(4), 585–594. https://doi.org/10.3233/NRE-230183

[35] Zhang, B., Wong, K. P., Liu, M., Hui, V., Guo, C., Liu, Z., Liu, Y., Xiao, Q., & Qin, J. (2025). Effect of artificial intelligence-based video-game system on dysphagia in patients with stroke: A randomized controlled trial. Clinical nutrition (Edinburgh, Scotland), 45, 81–90. https://doi.org/10.1016/j.clnu.2024.12.022

[36] Blázquez-González, P., Mirón-González, R., Lendínez-Mesa, A., Luengo-González, R., Mancebo-Salas, N., Camacho-Arroyo, M. T., Muriel-García, A., & García-Sastre, M. M. (2024). Efficacy of the use of video games on mood, anxiety and depression in stroke patients: preliminary findings of a randomised controlled trial. Journal of neurology, 271(3), 1224–1234. https://doi.org/10.1007/s00415-023-12043-z

[37] Sana, V., Ghous, M., Kashif, M., Albalwi, A., Muneer, R., & Zia, M. (2023). Effects of vestibular rehabilitation therapy versus virtual reality on balance, dizziness, and gait in patients with subacute stroke: A randomized controlled trial. Medicine, 102(24), e33203. https://doi.org/10.1097/MD.0000000000033203

[38] You, S. H., Jang, S. H., Kim, Y. H., Hallett, M., Ahn, S. H., Kwon, Y. H., Kim, J. H., & Lee, M. Y. (2005). Virtual reality-induced cortical reorganization and associated locomotor recovery in chronic stroke: an experimenter-blind randomized study. Stroke, 36(6), 1166–1171. https://doi.org/10.1161/01.STR.0000162715.43417.91

[39] Kwak, H. D., Chung, E., & Lee, B. H. (2024). The effect of balance training using touch controller-based fully immersive virtual reality devices on balance and walking ability in patients

with stroke: A pilot randomized controlled trial. Medicine, 103(27), e38578. https://doi.org/10.1097/MD.0000000000038578

[40] Sultan, N., Khushnood, K., Qureshi, S., Altaf, S., Khan, M. K., Malik, A. N., Mehmood, R., & Awan, M. M. A. (2023). Effects of Virtual Reality Training Using Xbox Kinect on Balance, Postural Control, and Functional Independence in Subjects with Stroke. Games for health journal, 12(6), 440–444. https://doi.org/10.1089/g4h.2022.0193

[41] Xiao, Y., & Xu, X. (2025). Enhanced stroke rehabilitation outcomes through Information-Motivation-Behavioral skills model and Hospital-Community-Family ternary linkage integration: A randomized controlled trial. Medicine, 104(9), e41547. https://doi.org/10.1097/MD.0000000000041547

[42] Ase, H., Honaga, K., Tani, M., Takakura, T., Wada, F., Murakami, Y., Isayama, R., Tanuma, A., & Fujiwara, T. (2025). Effects of home-based virtual reality upper extremity rehabilitation in persons with chronic stroke: a randomized controlled trial. Journal of neuroengineering and rehabilitation, 22(1), 20. https://doi.org/10.1186/s12984-025-01564-5

[43] Fluet, G., Qiu, Q., Gross, A., Gorin, H., Patel, J., Merians, A., & Adamovich, S. (2024). The influence of scaffolding on intrinsic motivation and autonomous adherence to a game-based, sparsely supervised home rehabilitation program for people with upper extremity hemiparesis due to stroke. A randomized controlled trial. Journal of neuroengineering and rehabilitation, 21(1), 143. https://doi.org/10.1186/s12984-024-01441-7

[44] Zhang, L., Yan, Y. N., Sun, Z. X., Yan, D. R., Chen, Y. W., Lin, K. C., Ge, X. J., & Qin, X. L. (2022). Effects of Coaching-Based Teleoccupational Guidance for Home-Based Stroke Survivors and Their Family Caregivers: A Pilot Randomised Controlled Trial. International journal of environmental research and public health, 19(23), 16355. https://doi.org/10.3390/ijerph192316355

[45] Kam Yuet Wong, F., Wang, S. L., Ng, S. S. M., Lee, P. H., Wong, A. K. C., Li, H., Wang, W., Wu, L., Zhang, Y., & Shi, Y. (2022). Effects of a transitional home-based care program for stroke survivors in Harbin, China: a randomized controlled trial. Age and ageing, 51(2), afac027. https://doi.org/10.1093/ageing/afac027

[46] Jarbandhan, A., Toelsie, J., Veeger, D., Bipat, R., Vanhees, L., & Buys, R. (2022). Feasibility of a home-based physiotherapy intervention to promote post-stroke mobility: A randomized controlled pilot study. PloS one, 17(3), e0256455. https://doi.org/10.1371/journal.pone.0256455

[47] Wolfe, C. D., Tilling, K., & Rudd, A. G. (2000). The effectiveness of community-based rehabilitation for stroke patients who remain at home: a pilot randomized trial. Clinical rehabilitation, 14(6), 563–569. https://doi.org/10.1191/0269215500cr362oa

[48] Mayo, N. E., Wood-Dauphinee, S., Côté, R., Gayton, D., Carlton, J., Buttery, J., & Tamblyn, R. (2000). There's no place like home : an evaluation of early supported discharge for stroke. Stroke, 31(5), 1016–1023. https://doi.org/10.1161/01.str.31.5.1016

[49] Ene, C. G., Gracey, F., & Ford, C. (2025). A feasibility randomized-controlled trial of an executive functioning telerehabilitation intervention for stroke survivors. Brain injury, 39(9), 772–783. https://doi.org/10.1080/02699052.2025.2483449

[50] Tsai, S. J., Li, C. C., & Pai, H. C. (2024). Effects of a nurse-led therapeutic conversations intervention in stroke patient-family caregiver dyads: A randomized control trial. International journal of nursing practice, 30(5), e13257. https://doi.org/10.1111/ijn.13257

[51] Mao, Y. R., Zhao, J. L., Bian, M. J., Lo, W. L. A., Leng, Y., Bian, R. H., & Huang, D. F. (2022). Spatiotemporal, kinematic and kinetic assessment of the effects of a foot drop stimulator for home-based rehabilitation of patients with chronic stroke: a randomized clinical trial. Journal of neuroengineering and rehabilitation, 19(1), 56. https://doi.org/10.1186/s12984-022-01036-0
