# Supplementary material for: A comparative study of five telerehabilitation therapies for improving core symptoms in stroke patients: A network meta-analysis (2,833 patients)
Source: iScience. 2026 Apr 17;29(6):115774. doi: 10.1016/j.isci.2026.115774 (PMC13196438; doi:10.1016/j.isci.2026.115774)
Supplement: Table S2. Evaluation results of literature quality risk bias of included articles [file mmc2.pdf]

Appendix 2. Evaluation results of literature quality risk bias of included articles

Inclusion of literature	Random sequence generation	Allocation concealment	Blinding of participants and personnel	Blinding of outcome assessment	Incomplete outcome data	Selective reporting	Other bias
Linder 2024	L	L	u	L	L	L	L
Rosenfeldt 2019	L	L	u	L	L	L	H
van de Port 2012	L	L	H	L	L	L	L
Rose 2011	L	L	L	H	L	L	H
Rodríguez-García 2025	L	U	L	U	L	L	u
Lu 2025	L	L	L	L	L	L	L
Kim 2025	L	L	L	L	L	L	L
Wang 2024	L	L	u	L	L	L	L
Liu 2023	L	L	L	L	L	L	L
Kim 2015	L	L	L	L	L	L	L
Jang 2016	L	U	H	L	L	L	u
Kasashima-Shindo 2015	L	U	L	L	L	L	u
Ramos-Murguialday 2013	L	L	L	L	L	L	L
Li 2014	L	L	u	U	L	L	u
Pichiorri 2015	L	L	H	L	L	L	L
Yu 2025	L	L	L	L	L	L	L
Wu 2025	L	L	L	L	L	L	L
Fong 2025	L	L	L	L	L	L	L
Ibolya 2025	L	L	H	L	L	L	L
Bustamante Valles 2016	L	L	L	L	L	L	L

Chang 2024	L	L	L	u	L	L	L
İrem 2024	L	L	L	L	L	L	L
Chen 2023	L	L	L	L	L	L	L
Wong 2022b	L	L	L	L	L	L	L
Zhang 2024	L	L	L	L	L	L	L
Huo 2024	L	L	L	L	L	L	L
Ho 2024	L	L	L	L	L	L	L
Song 2024	L	L	H	U	L	U	L
Jo 2024	L	L	H	L	L	L	L
Huang 2024	L	L	H	L	L	L	L
Cinakli 2023	L	L	L	L	L	L	L
Amin 2024	L	U	H	U	L	L	L
Aguilera-Rubio 2024	L	L	L	L	L	L	L
Rojas-Sosa 2023	L	U	L	L	L	U	u
Zhang 2025	L	L	u	L	L	L	L
Blázquez-González 2024	L	L	H	L	L	L	H
Vishal 2023	L	U	L	L	L	L	u
You 2005	L	L	L	L	L	L	H
Kwak 2024	L	L	u	U	L	L	L
Sultan 2023	L	L	L	L	L	L	L
Xiao 2025	L	L	L	L	L	L	L
Hiroyuki 2025	L	L	L	L	L	L	L

L：low risk of bias U: unclear risk of bias H：High risk of bias
